# Canine *Trichomonas tenax* mandibular gland infestation

**DOI:** 10.1186/s13028-016-0197-4

**Published:** 2016-02-18

**Authors:** Klaudiusz Szczepaniak, Anna Łojszczyk-Szczepaniak, Krzysztof Tomczuk, Tomasz Skrzypek, Barbara Lisiak, Zahrai Abd-Al-Hammza Abbass

**Affiliations:** 1 Department of Parasitology and Invasive Diseases, Faculty of Veterinary Medicine, University of Life Sciences in Lublin, at Ul. Akademicka 12, 20-033 Lublin, Poland; 2 Department and Clinic of Animal Surgery, Laboratory for Radiology and Ultrasonography, Faculty of Veterinary Medicine, University of Life Sciences in Lublin, at Ul. Głeboka 30, 20-612 Lublin, Poland; 3 Interdisciplinary Research Center, John Paul II Catholic University of Lublin, at Al. Kraśnicka 102, 29-718 Lublin, Poland

**Keywords:** Dog, Trichomonads, Salivary gland, Sialocele

## Abstract

**Background:**

Several species of trichomonads are intestinal or urogenital parasites of humans and animals, with only a few species typically being located in the oral cavity. The prevalence of oral trichomoniasis in dogs is approximately 15–25 %, although the prevalence varies among different populations and depends on age, sex and the health of the oral cavity.

**Case presentation:**

A case of mandibular gland infestation by trichomonads in a 13-year-old female Dachshund with advanced periodontal disease and oral trichomoniasis is reported. The dog was referred due to a history of a painless swelling over the left submandibular region that increased in size over time. Based on physical and ultrasound examinations, a final diagnosis of mandibular gland cyst was established and transcutaneous needle aspiration was carried out. Numerous mobile trophozoites of trichomonads were found by microscopy and culturing for trichomonas was performed. The species was finally characterized as *Trichomonas tenax* by polymerase chain reaction and sequencing.

**Conclusions:**

Studies have shown that *T. tenax* can be found in humans in atypical locations such as the salivary glands and upper and lower respiratory tracts. According to our knowledge this is the first report of *T. tenax* being present in the salivary glands of a dog. Because of the relatively high prevalence of trichomoniasis in dogs with periodontal diseases, these parasites should be considered together with bacterial and viral agents in salivary gland infections, especially in individuals with compromised oral health.

## Background

*Trichomonadea*, commonly known as trichomonads, comprises a class of amitochondriate flagellated protists belonging to the phylum *Parabasalia*. They are morphologically characterized by multiple anterior flagella and a single recurrent flagellum that functions as support for the undulating membrane [1]. Several species are considered to be intestinal or urogenital parasites of humans and animals. Only a few species are typically present in the oral cavity of animals [2] and to date, oral infestation caused by trichomonads in domestic carnivores have been reported rarely. The first report on oral trichomoniasis in dogs was done by Hegner and Ratcliffe [3], who described the species *Trichomonas canistomae.* Later, oral trichomoniasis was recorded by Beelitz et al. [4] and Breuker [5]. Recently, polymerase chain reaction (PCR) based molecular characterization of the various trichomonad species isolated from the oral cavity of dogs (mostly from Europe) confirmed the presence of *Trichomonas tenax*, *Trichomonas canistomae* and other *Trichomonas* sp., [6–9]. The prevalence of oral trichomoniasis in dogs is approximately 15–25 %, although the prevalence varies in different populations depending on age, sex and the clinical condition of the oral cavity [4, 5, 8].

Here we report the infestation of a salivary gland by *T. tenax* in a 13-year-old dog having advanced periodontal disease.

## Case presentation

**Fig. 1:**
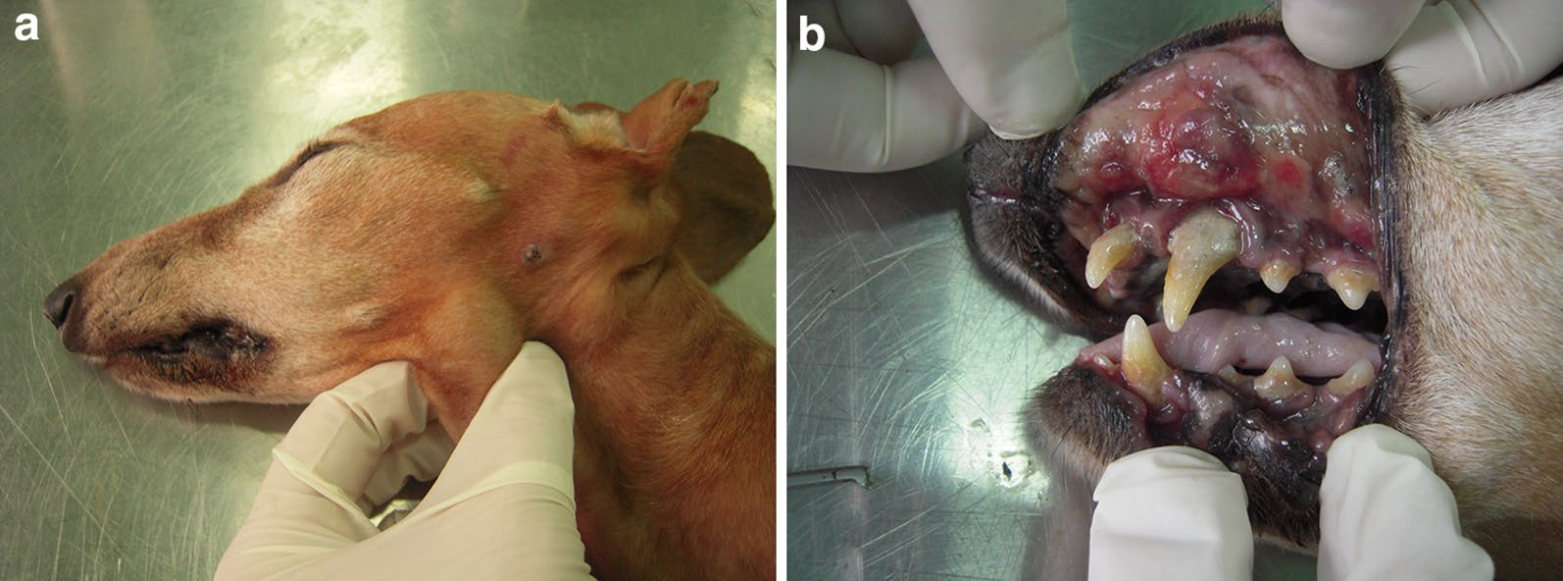
Clinical findings in a case of *Trichomonas tenax* infestation. **a** Swelling of the submandibular area. **b** Periodontitis and lesions of the oral mucosa

A 13-year-old female Dachshund was presented to the veterinary clinic at the University of Life Sciences in Lublin, Poland with a history of a soft painless oval-shaped swelling over the left side of the submandibular area that had gradually increased in size over time (Fig. 1a). The overlying skin was normal and there was no history of trauma or previous surgery in this region. Regional lymphadenopathy was not detected by palpation. According to the owner, the dog had suffered from persistent halitosis due to chronic periodontitis for several years. The dog had been treated with antibiotics or received enhanced treatment for periodontitis such as instrumental debridement and ultrasonic scaling several times during this period. An intraoral examination confirmed the existence of numerous lesions of the oral mucosa and advanced periodontal disease (Fig. 1b).

An ultrasound examination of the neck region was performed with the use of a linear 7.5 MHz transducer. The examination revealed the presence of an anechogenic cystic lesion in the left mandibular salivary gland measuring approximately 2.5 × 1.3 cm. Normal mandibular tissue was only partially visible. Changes were not observed in the right mandibular gland or in other neck tissues, including the lymph nodes, which were oval in shape and hypoechoic to the adjacent tissue.

Next, abdominal and thorax radiography was performed in dorsoventral and lateral recumbency, as well as abdominal ultrasonographic examination. These examinations did not reveal any significant changes. Based on the history and the physical and ultrasound examinations, a preliminary diagnosis of a mandibular gland cyst was established.

Transcutaneous needle aspiration of the left mandibular gland was carried out and a saliva-like fluid obtained. Microscopic evaluation of fluid revealed numerous, mobile trophozoites of trichomonads, mucus and aggregation of eosinophils. Swabs were also taken from gingival pockets and revealed the presence of trichomonads and a mixed bacterial flora.

The aspirated fluid was examined bacteriologically for aerobic and anaerobic growth and for yeast cultures but was found to be without growth.

The aspirated cystic content as well as swabs from gingival pockets were inoculated on a diphasic medium for cultivation of trichomonads: solid phase composition: 9 ml heat-inactivated horse serum, 1 ml 3 % beef extract, and 0.2 g glucose; liquid phase composition: 0.59 g Na_2_HPO_4_, 0.45 g KH_2_PO_4_, 8.0 g NaCl, dissolved in 1000 ml distilled water. The complete medium was allocated in sterile 10 ml tubes, supplemented with rice starch and 0.5 ml heat-inactivated horse serum. The media were cultivated at 37 °C and examined under light microscope every 24 h. Positive cultures were used for morphological characterization based on scanning electron microscopy. Trophozoites were prepared according to standard procedure for protozoa cells modified according to Szczepaniak [8]. In brief, trichomonads were isolated by centrifugation 2500×*g* for 5 min and fixed with 2.5 % glutaraldehyde in 0.1 M cacodylate buffer pH 7.2 for 2.5 h. Afterwards, the trichomonads were washed in phosphate-buffer saline and allowed to adhere to glass coverslips precoated with 0.1 % poly-l-lysine and post-fixed for 2 h with 1 % OsO4. Fixed samples were dehydrated in ethanol and acetone, dried in a critical point using a Polaron CPD 7501 critical-point dryer and coated with gold/palladium (Au/Pd) in a Polaron Range SC7620 sputter coater. Examinations were carried out using a ZEISS Ultra Plus scanning electron microscope.

Periodontal pocket swabs showed significant growth of trichomonads in the xenic culture in contrast to the cyst aspirate, which showed slow and poor growth. Undoubtedly, there are factors, which may influence the growth of trichomonads in vitro, such as low numbers of cells in the inoculum; but in the present study many trophozoites were found by direct microscopy of the aspirate. Because the aspirate was negative for bacteria and fungi, we hypothesise that the growth of trichomonads from the aspirate was compromised by the lack of bacteria. While some trichomonads such a *T. vaginalis* can be established directly into axenic cultures, others species (especially from alimentary track) require adaptation in monoxenic or polyxenic cultures, which is usually time consuming and laborious process [10, 11].

**Fig. 2:**
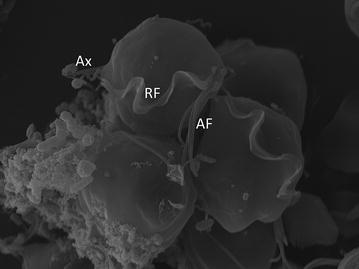
Scanning electron microscopy of the trophozoites of *Trichomonas tenax* isolated from the gingival pocket in a xenic culture. Note the four anterior flagella (AF), recurrent flagellum (RF) and the undulating membrane, axostyle (Ax)

Observation of trichomonads from a positive culture (5 days after inoculation) by light microscopy and scanning electron microscopy revealed numerous trophozoites and a single spherical, non-flagellated pseudocyst. The trophozoites varied in shape from piriform to spherical with four anterior flagella and a fifth recurrent undulating membrane and axostyle (Fig. 2). Accurate assessment of flagella size was difficult due to the large number of starch granules and bacteria. The major morphological features were typical for genera trichomonas or tetratrichomonas [12]. As detection and identification of trichomonads by conventional techniques are noticeably less reliable than PCR [12, 13], we chose to use molecular methods for taxonomical identification. The analyses were performed on the aspirate and the classification was based on the analysis of the partial sequence of the gene encoding in the SSU rDNA and the ITS region (ITS1, 5.8S rDNA, ITS2), after performing PCR with the primers TFR1 (5′-TGC TTC AGT TCA GCG GGT CTT CC-3′) and TFR2 (5′-CGG TAG GTG AAC CTG CCG TTG G-3′) according to the protocol described by Felleisen [9]. PCR products of approximately 370 bp were obtained from both samples (cystic content and gingival packet).

The DNA sequence was determined directly from the PCR products on both strands using the same primers employed for PCR at a DNA sequencing core facility (Genomed S.A., Warsaw, Poland). The obtained sequences were analysed using the Basic Local Alignment Search Tool program (BLASTn) and compared with those deposited at GenBank. The 368 bp fragment showed complete shared identity (100 %) with a sequence of *T. tenax* (strain—Hs-4:NIH,USA; ATCC 30207) with accession No. U86615.1

The owner decided not to bear the costs of treatment and the dog was euthanized by the wish of the owner. The dog was not necropsied.

## Conclusions

The most common salivary gland diseases in dogs are mucocele, sialocele and sialadenitis [14–16]. The underlying cause is often not established, but these conditions may be caused by blunt trauma, foreign bodies, sialoliths, autoimmune diseases or neoplasia. Canine sialadenitis has most often been related to bacterial invasions from the oral cavity as a secondary event to gingivitis/paradontosis or to specific viral diseases such as distemper or rabies [17, 18]. A parasitic aetiology of salivary disorders in dogs is uncommon; however, according to van der Merwe et al. [19] canine spirocercosis occurs frequently in dogs with salivary gland enlargement. The present study is apparently the first report of infestation of the salivary glands by *T. tenax* in a dog.

*Trichomonas tenax* is considered specific to humans and knowledge on its zoonotic potential is limited [20]. However, the isolation of *T. tenax* from various domestic animals such as dogs, cats and horses sheds new light on its narrow specificity and host range [6, 8]. Opinions about the pathogenicity of *T. tenax* are divided. It is frequently found in humans with poor oral hygiene and in individuals with advanced stages of periodontal disease [21]. A significant correlation between the occurrence of *T. tenax* and periodontal diseases has been demonstrated in dogs [8]; however, the roles of *T. tenax* in canine periodontal disease is still not fully understood. Proteolytic and collagenolytic activities as well as cytotoxicity abilities of *T. tenax* were observed in in vitro studies, the results suggest, that this organism should be considered more as parasite than a commensal of oral cavity [22].

Although, *T. tenax* has a tropism for the oral cavity, infections in other locations such as salivary glands [23], lymph nodes [24] and upper and lower respiratory tracts [25–28] have been reported. Such infections usually tend to be associated with concurrent diseases or with immunosuppression due to long-term corticosteroid therapy. In the present case, the only additional condition seen was a chronic periodontitis and it is likely that the infestation of a mandibular gland by *T. tenax* was secondary to the oral infection with *T. tenax* found in gingival pockets. Because of the relatively high prevalence of trichomonads in dogs with periodontal diseases, these parasites should be considered as a potential aetiological agent in salivary gland infections, especially in individuals with poor oral hygiene.
